# Deep learning can automate chicken tibia-breaking strength quantification to improve animal welfare

**DOI:** 10.1016/j.psj.2026.106549

**Published:** 2026-01-30

**Authors:** Tanmay Debnath, Peter Wilson, Ricardo Pong-Wong, Lindsey Plenderleith, Björn Andersson, Matthias Schmutz, Ian Dunn, James G.D. Prendergast

**Affiliations:** a The Roslin Institute, University of Edinburgh, Easter Bush, Midlothian EH25 9RG, UK; b Lohmann Breeders GmbH, 27472 Cuxhaven, Germany

**Keywords:** Deep learning, Keel, Bone strength, X-ray

## Abstract

Bone damage is an important welfare issue in the poultry industry, yet large-scale phenotyping of chicken bone strength currently relies on time-consuming manual annotation of X-rays or destructive post-mortem testing. To address this, an end-to-end deep-learning pipeline was developed that automatically (i) segments the chicken tibiotarsus from lateral X-ray images (U-Net, Dice = 0.91) and (ii) predicts its breaking strength from pixel intensities alone. Using 916 curated bone images, the predictor achieved moderately high correlation with measured breaking strength (maximum Pearson’s correlation of 0.74), exceeding the performance of a previous labour-intensive manual annotation method. Image-derived predictions were moderately heritable (h² ≈ 0.16) and exhibited an exceptionally high genetic correlation with the physical trait, indicating that selection on the model-derived phenotype is a good proxy to select for bone strength. The workflow therefore provides a potential rapid, non-invasive and genetically informative alternative to post-mortem testing, paving the way for the routine incorporation of bone-quality traits into commercial breeding programmes and improved poultry welfare at scale.

## Introduction

Surveys across Europe and elsewhere show that many consumers are aware of welfare issues in egg production and are willing to pay more for products perceived as higher welfare (Gautron et al., 2021). As a consequence, to improve the welfare of chickens, more extensive housing systems have been introduced that, as an added benefit, enhance chicken mobility and increase mechanical loading on the skeleton, which results in stronger bones (Rodriguez-Navarro et al., 2018). However, these systems are often associated with higher incidences of bone damage (Sandilands, 2011; Stratmann et al., 2015), especially for the keel (Maidin et al., 2025; Rørvang et al., 2019), although this is not true for every system studied (Rufener and Makagon, 2020). Consequently, once hens are out of cages, bone fractures become one of the primary welfare concerns (FAWC, 2010; Nasr et al., 2012; Toscano et al., 2020), with physiological adaptation for egg laying further increasing the likelihood of animals suffering from osteoporosis (Whitehead, 2004). Specifically, the transition to the laying state is accompanied by a shift to the laying down of medullary bone, which acts as a labile source of calcium for egg shell formation, meaning structural cortical bone formation is diminished (Dacke, et al., 1993; Gautron et al., 2021).

Bone strength in chickens, i.e. how much load a bone can withstand before it fails, has been repeatedly found to be a heritable trait (Andersson et al., 2023; Bishop et al., 2000; Dunn et al., 2021; Raymond et al., 2018). A key challenge to improving bone strength via selective breeding is that the gold-standard quantification of breaking strength has to be done post-mortem, after the bone is dissected for further analysis (Fleming et al., 1994), an approach which has been used experimentally to create lines and ultimately F2 populations by retrospective selection (Bishop et al., 2000; Dunn et al., 2007). This is a key barrier to implementation in commercial breeding programs. Thus, a non-invasive proxy measurement was previously developed that can characterise bone quality parameters in live birds in terms of their density, and these were observed to be correlated with previously established post-mortem analyses of bone strength (Wilson et al., 2023). Other non-invasive methods include dual-energy X-ray absorptiometry or DEXA (Schallier et al., 2019), computed tomography (Korver et al., 2004), digitised fluoroscopy and ultrasound (Fleming et al., 2004). However, advancements in digital radiography mean it has become a viable option for greater simplicity and speed of data acquisition (Körner et al., 2007). Thus, large-scale X-ray imaging of live birds is being explored in the poultry community (Andersson et al., 2023; Jung et al., 2022; Rufener et al., 2018). Ultimately, all measures are seeking ways of defining quality factors that will reduce bone fractures.

With the advancement of computational methods, such as deep learning, analysing images in bulk has become fast, reliable and efficient. Deep learning forms a class of algorithms that are efficient at extracting high-level, abstract features from raw data and, thus, enables a computer to perform complex tasks with limited intervention (Goodfellow et al., 2016). Recently, deep learning methods have seen many applications in the X-ray imaging domain. Examples range from binary classification, where the main task of the algorithm was to classify an X-ray image to be positive for a disease or not (Chidziwisano et al., 2025), to segmentation, where the algorithm was tasked to extract specific sections from the image (Çallı et al., 2021). More specifically, they have been utilised to analyse X-ray image datasets to detect COVID-19 (Rajaraman et al., 2020; Tartaglione et al., 2020), pneumothorax (Jennie et al., 2020; Wang et al., 2020), tuberculosis (Hwang and Kim, 2016; Pasa et al., 2019), and several other diseases. Similarly, these algorithms have also been explored in modern veterinary diagnostics and animal health (Xiao et al., 2025). Applications include understanding canine maturity and bone fracture time (Ergün and Güney, 2021), detecting spinal cord diseases (Biercher et al., 2021), predicting chicken breast muscle weight *in vivo* (Zhu et al., 2024), and more recently, identifying the chicken keel (Sallam et al., 2024). This proves that deep learning methods can be used to understand the underlying features of various datasets and might help us create better diagnostic systems, thus promoting animal health and, in our case, providing quantitative data to improve bone quality.

Keel bone damage is one of the major chicken welfare concerns in laying hens, and it has been previously explored to understand the bone mineral quality in birds (Habig et al., 2021; Riber et al., 2018). However, due to the difficulty of examining the keel bone in X-ray images due to overlapping muscle and feather coverage, examination of the tibia has been suggested as a useful proxy to define general bone strength (Bishop et al., 2000; Wilson et al., 2023). Classification of the damage to the keel relies on there being an interaction with chance events, namely collisions or behaviour leading to breaks or deformation. Recent work has demonstrated that there is a significant relationship between keel damage and tibia tarsus quality, where the keels with the worst scores, representing severe keel bone damage, had lower tibia breaking strengths and density than those with a low damage score (Maidin et al., 2025). Interestingly, the keels with the most fractures and deviations had the highest radiographical density, which is due to callus formations on damaged keels (Maidin et al., 2025). Consequently, the direct assessment of keel density can be problematic as it is confounded by both overlapping tissues and the results of damage, but using morphology, it has been achieved (Duenk et al., 2025). To make measurements practical and usable for animal breeding, we tested the hypothesis that a deep learning model could predict the tibiotarsal breaking strength from X-ray images only. We highlight that our model predictions are genetically highly correlated with post-mortem tibia-breaking strengths, thus confirming their potential for use in animal breeding.

## Materials and methods

**Fig. 1:**
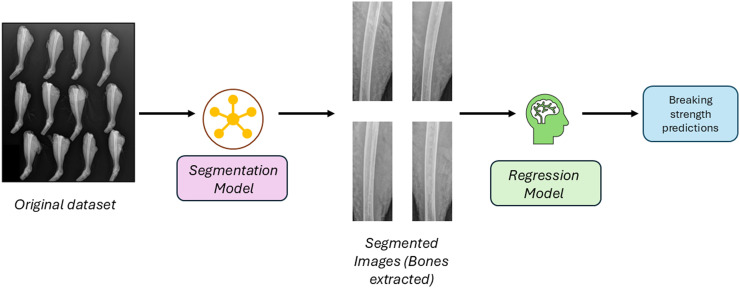
The overall pipeline for predicting bone strength from X-ray images. The pipeline can extract the bones from the stack of bones in a single image and later use the extracted images to read the pixel information to predict the bone strengths.

In this research, an image extraction method was developed using a segmentation model that can first isolate the bone from the wider image. These extracted images were then used to train models to predict the tibia-breaking strength (shown in Fig. 1).

### Animal population and imaging dataset

The dataset was derived post-mortem from White Leghorn hens (*n* = 952), 100 weeks of age, from a line that has previously been studied (Andersson et al., 2023). The hens were a pure grandparent line of Lohmann LSL commercial layers (Lohmann Breeders GmbH, Germany), comprising 53 sires and 434 dams. Normally, this type of X-ray imaging would be performed on live chickens. However, in this instance, due to COVID-19 restrictions, the dataset was collected by imaging freshly dissected legs. The X-ray equipment used consisted of a Cuattro Slate 6 DR X-ray system with a Wireless 12 × 14″ AED VW Caesium Wireless Flat Panel Detector (100um pixel). X-rays were generated using a MeX +20BT lite Battery-Powered X-ray Generator (set at 65 kV/ 5 mAs), suspended from a Stat-X Vaquero Folding Mobile Stand 100 cm above the detector (IVM Imaging, Bellshill, Scotland). The dataset comprised a total of 80 radiographs, each comprising freshly excised complete right chicken legs that were positioned in a fixed 3 × 4 matrix, giving the 952 total (79 images × 12 bones and 1 image × 4 bones) (shown in Supplementary Fig. S1). Tibia breaking strength was measured by a three-point bending test using a material testing machine fitted with a 2.5KN load cell (AMETEK LS5, Sussex, UK) using Nexygen plus software, but otherwise similar to that described previously (Fleming et al., 1994). The area under the curve (AUC) measurement for each of the tibiae was made as described previously (Wilson et al., 2023). In brief, a fixed rectangular selection was made across the mid-point of the tibiotarsus, and the mean pixel density of the area along its cross-section was calculated. In such X-ray based analyses, pixel intensity is essentially a proxy for how much X-ray was absorbed by different tissues, which in turn reflects tissue thickness, composition and density. So this AUC approach is effectively measuring how much dense, X-ray absorbing material lies across that cross-section, that is expected to reflect the total mineralised cross-sectional area. The higher the pixel density across the transect, the denser the bone.

The descriptive statistics of the features explored in this study can be observed in Table 1. The mean of the measured tibia breaking strength was ≈ 225 N, with a median of ≈ 212 N and a standard deviation of ≈ 74 N. Similarly, it could be observed that the tibia AUC scores have a mean of ≈ 84000, with a median of ≈ 82700 and a standard deviation of ≈ 10000.

**Table 1:** The descriptive statistics table. The table shows the distribution of data across our dataset.

Features	Mean	Median	Standard Deviation	Minimum value	Maximum value
Measured Tibia breaking strength	224.88	212.31	73.73	66.53	697.31
Tibia AUC scores	83591.54	82725	9667	61039	135489

### An overview of the segmentation task

#### Data Pre-processing for Segmentation

All the radiographs were passed onto the segmentation module to extract the candidate tibiae. The LabelMe software package (Russell et al., 2008) was used to manually annotate a mask of the tibia for model training and validation.

#### Overview of the segmentation model

Before a regression model could be trained, individual tibiae had to be cropped from their parent radiographs, which would be the output from the segmentation model. A CNN (Convolution Neural Network) based model was chosen to be appropriate for the task. The U-Net segmentation model (Ronneberger et al., 2015) was implemented to distinguish the bone from the background and soft tissue. The model went through each pixel in the image and classified whether the pixel was associated with the background (which includes soft tissue and other anatomical regions) or the tibia bone. It was used for its wide applicability in a range of different X-ray image-based segmentation tasks (Krithika alias AnbuDevi and Suganthi, 2022). It is a 23-convolution-layered U-shaped encoder-decoder network architecture, which consists of four encoder blocks and four decoder blocks that are connected via bridge connections (shown in Supplementary Fig. S2).

The model took 256 × 256 pixel images as input. The encoder section mainly consisted of 3 × 3 convolution networks, followed by a ReLU (Rectified Linear Unit) layer and a 2 × 2 max pooling operation with a stride of 2 for downsampling. It should be noted that at each downsampling stage, the total number of feature channels increased. Whereas, in the decoder section, an upsampling of the feature map followed by a 2 × 2 convolution layer could be observed. After upsampling, two 3 × 3 convolution operations were added and concatenated with the corresponding layer from the contracting side. At the final layer, a 1 × 1 convolution was used to map these 64-component feature vectors to a desired number of classes, which is 1 in our case (the bones and the background).

At the end of the segmentation process, a rectangular region enclosing the segmentation was extracted, as it provided the model more context about the surroundings and thus ensured richer information to extract from. These images were then cropped to 200 pixels horizontally by 500 pixels, where the long axis was parallel to the central tibia bone, which comprises the cortical bone and the medullary bone it encompasses, thus making the images consistent in terms of size for the next steps (shown in Supplementary Fig. S1). All of these modifications were done automatically using a Python script.

### An overview of the regression task

#### Data pre-processing for regression

After segmentation, a manual inspection was conducted on the segmentation results. In total, 36 images were removed based on segmentation errors, missing wingbands and family structure information. A total of 916 images were finally selected for the regression analysis, retaining 96% of the original dataset.

#### Overview of the regression model

For the regression models, akin to the segmentation model, CNN-based models were chosen. A ResNet-based model (He et al., 2015) was employed for its efficient training and excellent feature extraction capabilities.

Residual networks have helped us train deeper networks. Each block in the ResNet-50 model (shown in Supplementary Fig. S3) consists of three convolution layers with batch normalization and ReLU activation after each of the layers. Each block started with a convolution layer of filter size 1 × 1 to reduce the number of channels from the previous layer. In the feature extraction layer, a convolution layer of filter size 3 × 3 was used to extract spatial features from the data. Then, to the final layer of the block, another convolution layer of 1 × 1 filter size was added to restore the original number of channels before the output was added to the shortcut connection (or skip connection). Because of the presence of these skip connections, deeper models could be trained without facing the vanishing gradient problem (He et al., 2015). The total parameter size of the ResNet-50 model is ∼23.51 million.

### Hyperparameter search and model training

The hyperparameters for the model training were optimised using Optuna (Akiba et al., 2019) for both segmentation and regression models.

#### Segmentation model

The hyperparameters for the segmentation model comprise the learning rate, weight decay and the kind of optimizer. In the optuna fine-tuning scheme, a total of 300 trials were conducted. For each trial, the dataset was randomly split into training and validation sets, respectively. About 80% of the dataset was allocated to the training set (64 images; 760 legs), and the remaining 20% of the dataset was allocated equally to the validation and test datasets (16 images; 192 legs). The model was trained for 250 epochs per trial and then tested on the testing set. The dice loss (Sudre et al., 2017) was used as the loss function for all of the trials. No regularization was introduced because of the inherent stability of the process.

For training the final segmentation model, the model was trained in a distributed training fashion on 2 NVIDIA A100 GPUs. The model was trained across 5 trials. For each trial, the training dataset had 70% of the complete dataset (56 images), and the remaining 30% of the dataset was split equally between the testing and validation datasets (24 images). For training the model, we have used a dice-loss-based loss function as shown below:

$$\text{diceloss}=1-\frac{prediction\cap labels}{prediction\cup labels}$$

The main function of the dice loss is to ensure that the model is optimised based on the dice coefficient, which is a measure of the accuracy of the segmentation prediction. We have used the AdamW optimizer (Loshchilov and Hutter, 2019) for model training, with $1.91\;\times 10^{-5}$ and $1.05\;\times 10^{-8}$ as its learning rate and weight decay, respectively. The model was trained for a total of 250 epochs in each trial.

#### Regression models

For training our regression models, a k-fold cross-validation strategy was incorporated, as shown in Supplementary Fig. S4, where hyperparameters for the model are chosen during the model training itself. The entire training pipeline can be split into different segments for easier understanding.

Firstly, the data was split into training and testing datasets. Throughout the training process, the model was unaware of the existence of the testing dataset, and only the training dataset was used to train and validate the model. More importantly, the same testing dataset was used for all of our analyses. About 15% of the total dataset (139 legs) was assigned to the testing dataset, and the rest to the training dataset (777 legs). To find the hyperparameters for the model training, Optuna was used, and a 5-fold cross-validation strategy was employed. In this strategy, Optuna suggests a set of hyperparameters, and based on the model performance across multiple trials, a final set of hyperparameters was chosen for final model training. Initially, the training dataset was split into 5 folds. The model was trained on any 4 of the folds at a time and validated on the remaining one. On the validation fold (left-out fold), the model performed inference and predicted the corresponding tibia breaking strength for the given image sample. Based on the predictions, Pearson’s correlations between the model predictions and the measured tibia breaking strengths were calculated. This correlation was stored until every fold became a part of the validation dataset once, thus giving us 5 correlations from each fold. To estimate whether the set of hyperparameters was good, the mean of the correlations was taken as the metric. This gave us a measure of how good the model performance was across different folds, thus not relying too much on a particular fold's information and forcing the model to generalise its knowledge. The process was performed for t trials (t=20 for our experiments), and the set of hyperparameters that gave us the highest mean correlation was chosen. This set of hyperparameters was used for the final model training for n epochs (n=50 for our experiments). Finally, the trained model was tested on the testing dataset that was initially separated.

### Model explanation

Understanding which regions of the bone were important to a model can explain the functioning of the model. To do so, the gradients of weights in the model layers were observed. The gradient associated with a model weight indicates the amount of update that particular weight was undergoing for a given backward pass. This, in turn, indicates the importance of that weight in the model’s final predictions. Thus, a weight undergoing higher updates would probably have a higher effect on the final prediction than one with relatively lower updates. In our case, the gradients were normalised between 0 and 1 to observe the proportion of changes happening in the image. To obtain the associated gradients, an image was selected at random from the test dataset to observe the model's behaviour for novel data. Next, a forward pass of the image tensor was done, and then the gradients were aggregated across different layers in the next backward pass. Finally, the aggregated gradients were normalised to observe the proportion of weight changes happening in different image regions.

A glossary table of common deep learning model-related terms used in this study has been added as supplementary information (see Supplementary Glossary).

### Genetic correlation analysis

To understand if our model could learn any meaningful insight into the genetic control of the trait from the images, a bivariate genetic analysis was conducted. The genetic correlation between the measured tibia breaking strength and the AI model’s predictions was calculated, using pedigree information. The genetic correlations between the traits were analysed using a bivariate linear mixed model in ASReml (A R Gilmour, 2021). The bivariate model could be represented as:

$$\left(\begin{array}{c} Y_{1}\\ Y_{2} \end{array}\right)=\left[\begin{array}{cc} X_{1} & 0\\ 0 & X_{2} \end{array}\right]\left(\begin{array}{c} b_{1}\\ b_{2} \end{array}\right)+\left[\begin{array}{cc} Z_{1} & 0\\ 0 & Z_{2} \end{array}\right]\left(\begin{array}{c} u_{1}\\ u_{2} \end{array}\right)+\left(\begin{array}{c} e_{1}\\ e_{2} \end{array}\right)$$

Where, $Y_{1}$ and $Y_{2}$ are the vectors of phenotypic records for trait 1 (measured tibia breaking strength) and trait 2 (AI predicted breaking strengths), respectively; $b_{1}$ and $b_{2}$ represents the fixed effects of both traits; $u_{1}$ and $u_{2}$ represents the random effects associated with both traits; $e_{1}$ and $e_{2}$ represents the residuals for both traits. Here, $X_{1}$ and $X_{2}$ represents the design matrices for fixed effects in both traits; $Z_{1}$ and $Z_{2}$ represents the design matrices for the random effects in both traits. These matrices relate the observations for our traits with their effects. The random effects and residuals are sampled from:

$$\left(\begin{array}{c} u_{1}\\ u_{2} \end{array}\right)∼N\left(0,\left(\begin{array}{cc} A\sigma_{u_{1}}^{2} & A\sigma_{u_{1,2}}\\ A\sigma_{u_{1,2}} & A\sigma_{u_{2}}^{2} \end{array}\right)\right)\text{and}\;\left(\begin{array}{c} e_{1}\\ e_{2} \end{array}\right)∼N\left(0,\left(\begin{array}{cc} I\sigma_{e_{1}}^{2} & I\sigma_{e_{1,2}}\\ I\sigma_{e_{1,2}} & I\sigma_{e_{2}}^{2} \end{array}\right)\right)$$

Where, A is the relationship matrix calculated from the pedigree of animals; I represents the identity matrix. Here, $\sigma_{u_{1}}^{2}$ and $\sigma_{u_{2}}^{2}$ represents the genetic variance associated with traits 1 and 2, respectively; $\sigma_{u_{1,2}}$ represents the genetic covariance associated with the traits. Similarly, $\sigma_{e_{1}}^{2}$ and $\sigma_{e_{2}}^{2}$represent the residual variance associated with the traits; $\sigma_{e_{1,2}}$represent the residual covariance between the traits. Now, upon simplifying the bivariate model into individual univariate models yield:

$$y_{1}=X_{1}b_{1}+Z_{1}u_{1}+e_{1}$$

$$y_{2}=X_{2}b_{2}+Z_{2}u_{2}+e_{2}$$

Thus, the individual heritability of these traits could be described as:

$$h_{1}^{2}=\frac{\sigma_{u_{1}}^{2}}{\sigma_{u_{1}}^{2}+\sigma_{e_{1}}^{2}}$$

$$h_{2}^{2}=\frac{\sigma_{u_{2}}^{2}}{\sigma_{u_{2}}^{2}+\sigma_{e_{2}}^{2}}$$

And, the genetic correlations between these traits could be described as:

$$r_{g}=\frac{\sigma_{u_{1,2}}}{\sqrt{\sigma_{u_{1}}^{2}\sigma_{u_{2}}^{2}}}$$

### Training pipeline for genetic correlation analysis

Similar to the regression model training methods, a k-fold cross-validation strategy was incorporated, as shown in Supplementary Fig. S5. Here as well, the model hyperparameters were chosen during the model training phase itself.

Firstly, the data was split into 5 folds, where one fold is taken as the testing fold (as shown in Supplementary Fig. S5 as ‘Test F’) and the rest of the folds were combined to form the training dataset (named as ‘Total training folds data’). Similar to our training methodology, a 5-fold cross-validation analysis was performed to find the best hyperparameters. Optuna was used here to search for the hyperparameters over t trials (here, t=20 for our experiments). For a given set of hyperparameters, our method performed the 5-fold cross-validation and collected the Pearson’s correlations for each of the folds left out during each cross-validation analysis. The mean of the correlations was taken as the metric to characterise the effectiveness of the set of hyperparameters for the given dataset. After t trials, the set of hyperparameters with the highest mean correlation was chosen and used to finally train the model on the full training dataset. The trained model was then used to predict on the testing dataset. The model’s predictions were stored in computer memory. This process was continued until all the training folds (mentioned as ‘Train F. 1′, ‘Train F. 2′, ‘Train F. 3′, and ‘Train F. 4′ in Supplementary Fig. S5) became a part of the testing fold once. During this entire process, the model’s final predictions were stored in computer memory and concatenated with the previous iteration’s predictions. The final dataset was then saved as a CSV file, which was later used for the genetic correlation analysis.

## Results

### Segmentation model for automated bone isolation

Using a dataset of X-ray images of the right legs of 952 White Leghorn chickens, we first explored the ability to use a CNN to isolate the tibia bones. A dice coefficient (the measure of similarity between the predicted segmentation mask of the bone and the manually annotated segmentation mask) of ∼0.91 was achieved on the held-out test dataset across different trials. This indicates a high overlap between predicted and ground-truth masks. Inference with the optimised U-Net on all 80 radiographs produced 949 putative bone masks and their enclosing bounding boxes.

### Prediction of breaking strength from images

We next explored the ability to predict bone breaking strengths from these segmented images of the tibia bone. To translate pixel intensities into continuous estimates of tibiotarsus breaking strength, we adopted ResNet-50, a 50-layer convolutional neural network.

**Fig. 2:**
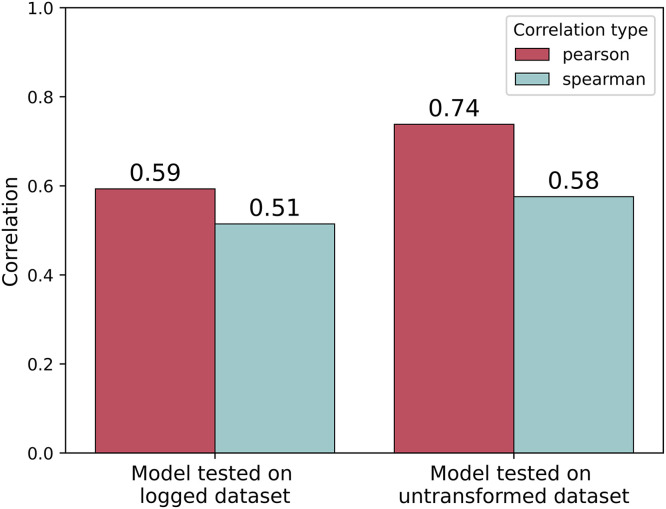
Model performance on the test data. The model was cross-validated to find the best possible correlations between the predictions and the actual breaking strengths for both the untransformed and natural log-transformed data. The cross-validated model was later used to make predictions on the test dataset.

As 24 birds had unusually high tibia-breaking strengths (greater than 2.58 SD above the mean of the dataset), we fitted two parallel models. In one, the model was trained on the actual tibia-breaking strengths on their original scale (referred to as the ‘untransformed’ dataset), whereas in the other, we trained the same model on the natural log-transformed tibia-breaking strengths to down-weight the contribution of the extreme values. From Fig. 2, it can be observed that the model predictions had good positive correlations with measured mechanical strength in both cases. The larger gap between Spearman’s and Pearson’s correlations for the model trained on the raw data (Spearman’s correlation: 0.58; Pearson’s correlation: 0.74) indicates potential sensitivity to extreme individuals. However, although applying a log transform reduced this difference by 22%, the general performance was higher for the untransformed model in both cases.

The ability of a single CNN to explain a proportion of the variance in an unseen test set demonstrates that pixel-level information alone captures a substantial portion of the determinants of tibiotarsus strength.

### The model performs on par with the previous manual method

**Fig. 3:**
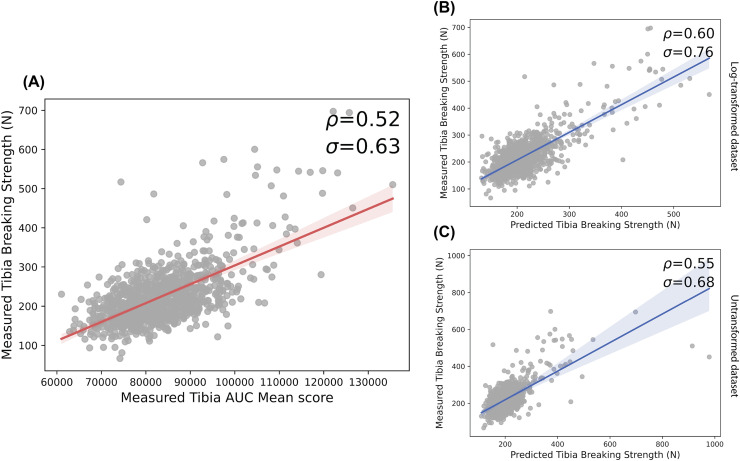
The model performance compared to the mean AUC score method and actual breaking strengths for the entire dataset (ρ: Spearman’s correlation; σ: Pearson’s correlation). The model was trained according to our training scheme across different folds. (A) The correlation between measured tibia breaking scores and the AUC mean scores. (B) The correlation between measured tibia breaking strength and our model’s predictions when the model was trained on the untransformed dataset. (C) The correlation between the measured tibia breaking strength and our model’s predictions when the model was trained on the natural logged dataset.

In a previous approach, the AUC (area under the curve) in terms of pixel intensity associated with a manually-annotated transect of the tibia bone was shown to be an effective correlate of recorded breaking strength (Wilson et al., 2023). Fig. 3 shows the relative performance of our models and the AUC method at predicting tibia-breaking strengths when the methods were tested specifically on the whole dataset.

The overall correlation of the measured tibia breaking strength with the mean AUC score was measured (Spearman’s correlation, ρ: 0.52; Pearson’s correlation, σ: 0.63; *p* < 0.001) (as shown in Fig. 3A). The predictions from the model trained on untransformed data were more highly correlated with the AUC values (Spearman’s correlation, ρ: 0.60; Pearson’s correlation, σ: 0.76; *p* < 0.001) than the model trained on log-transformed data (Spearman’s correlation, ρ: 0.55; Pearson’s correlation, σ: 0.68; *p* < 0.001).

Importantly, it could be observed that the deep-learning approach exceeded the accuracy of predicting measured breaking strengths over the established AUC proxy (Fig. 3B, C) with maximum Spearman’s and Pearson’s correlations of 0.60 and 0.76, respectively.

### Explaining model layers

**Fig. 4:**
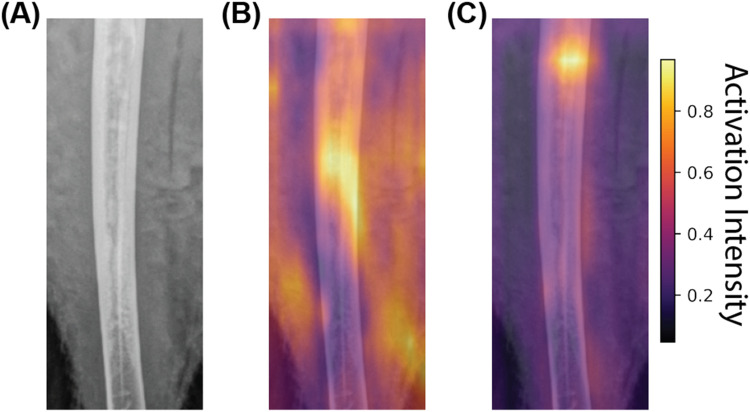
An overview of the model explanation results for the regression model. The model, through different layers, captures different aspects of the images, whose combined knowledge leads to the final prediction. (a) The original image used from the test dataset. (b) The cumulative gradient of weight changes associated with the input tensor across different layers of the ResNet-50 model when the model is trained on the unaltered dataset. (c) The cumulative gradient of weight changes associated with the input tensor across different layers of the ResNet-50 model when the model is trained on the log of the tibia-breaking strength dataset.

Fig. 4 shows the gradient changes in different regions for a given image. From the gradient maps, as shown in Fig. 4B and Fig. 4C, it could be observed that the untransformed model is not only using pixels at the bones but also from the surrounding muscles to make a prediction. However, following the logging of the input dataset (transformed dataset), the model focused more on a very specific location of the bone. These data suggest the model predictions reflect underlying biology.

### Model predictions can be used as an effective metric for selection

To understand if our model predictions have merit for breeding programs, a bivariate genetic analysis was conducted between predicted tibia breaking strengths and measured tibia breaking strengths. Key variance-component estimates are summarised in Table 2, Table 3. The CNN-derived phenotypes are moderately heritable (h² ≈ 0.15 and 0.16) as compared to the measured phenotypes (h² ≈ 0.23), indicating that genetic variance captured by the images can be transmitted across generations. Most critically, the genetic correlation between the image-based scores and true breaking strength is extremely high ($r_{g}$ ≥ 0.90). This suggests that the model has captured the genetic variance that influences the measured tibia breaking strengths.

**Table 2:** The genetic correlation analysis of unaltered tibia-breaking strengths and the corresponding model predictions. For this case, we have used the actual tibia-breaking strengths for model training.

	Heritability (h^2^)	Genetic correlation
Tibia-breaking strength (N)	0.23 ± 0.07	0.99 ± 0.05
Model Predictions	0.16 ± 0.06

**Table 3:** The genetic correlation analysis of the logged tibia-breaking strengths and the corresponding model predictions. For this case, we have used the logged tibia-breaking strengths for model training.

	Heritability (h^2^)	Genetic correlation
Tibia-breaking strength (N)	0.23 ± 0.07	0.90 ± 0.1
Model Predictions	0.15 ± 0.06	

## Discussion

In this research, we explored how deep learning methods could be implemented to identify tibias from chicken X-ray images and predict their corresponding breaking strengths. Well-established off-the-shelf architectures have been used, which indicates that such methods can be used to extract and interpret the composition of pixels in the images. Based on that information, informative predictions of utility could be made to breeding programs, as the genetic correlation between the image-based predictions and the data gathered from actually breaking the bones was greater than 0.9.

While our workflow is mostly automated, certain data-cleaning steps were performed manually. Although the number of images involved was comparatively small, if this workflow were to be implemented at scale in a production environment, automated quality controls of segmentation results would be required.

While increasing sample size would almost certainly improve the stability and generalisability of the CNN, several additional strategies could be used to optimise model performance. For example, data augmentation could be applied to better mimic *in vivo* imaging variability, including controlled rotations, translations, scaling, and contrast or noise perturbations. This would encourage the model to become invariant to positioning and acquisition differences that may be more common in live bird imaging, and may reduce overfitting to the highly standardised post mortem setup used here. Though with proper control of bird positioning the advantages of this may be limited.

Likewise, architecture and loss function optimisation could be explored. Although ResNet 50 provided a strong baseline, alternative backbones or architectures that explicitly model long-range context may capture additional spatial relationships relevant to bone strength. Transferring learning from models pre-trained on large medical X-ray datasets may also prove to be beneficial, particularly when moving from post-mortem imaging to *in vivo* radiographs with different noise characteristics. Explicitly modelling uncertainty in the predictions (e.g. via Monte Carlo dropout or deep ensembles) could help identify images for which the model is unreliable, enabling targeted re-imaging or exclusion from genetic evaluations, and thereby improving the effective accuracy of selection on the CNN-derived phenotype.

Beyond pure image-based optimisation, future work could investigate multimodal models that combine radiographic information with readily available covariates (such as body weight, age, or housing group). If such covariates capture systematic environmental effects rather than genetic differences, including them could reduce residual variance in the CNN phenotype and thus increase both its heritability and the precision of estimated genetic correlations, even without a large increase in image numbers.

We wanted to explore whether the CNN predictions were able to capture the genetic variation within the population. To understand the same, the genetic correlation between the model’s predictions and the tibia-breaking strengths was estimated. The models’ predictions, in both the untransformed dataset and the logged dataset, showed similar heritabilities. Importantly, our model predictions have a high and positive genetic correlation with the tibia-breaking strengths. This suggests that, for both log-transformed and untransformed datasets, the models are capable of extracting important features from the images and making predictions that have a genetic correlation greater than 0.90 with the observed values. The values of heritability are lower than previously published (Dunn et al., 2021), but this is likely related to the age of the birds and was similar to values obtained previously for these hens (Andersson et al., 2023). The heritability decreased with age from around 0.62 to around 0.28, we believe because a number of birds spent longer periods not laying eggs. Cumulatively, this confirms that our model can potentially be used as a tool for selecting chickens based on X-ray images only, with an aim for breeding for improved bone strength.

There are some examples of models used in other animal studies for tasks such as estimating canine skeletal maturity, classifying spinal cord lesions, or predicting broiler breast muscle weight from radiographs and photographs (Ergün and Güney, 2021; Biercher et al., 2021; Zhu et al., 2024). However, most such applications focus on classification or ordinal scoring rather than direct prediction of an underlying biomechanical property, and we think that comparing metrics between studies with different goals and types of training data could be misleading. To our knowledge, this is, though, one of the first animal studies to show that a CNN trained solely on conventional radiographs can predict an experimentally measured mechanical strength trait with a correlation approaching 0.75 and a genetic correlation with the true trait exceeding 0.9, underscoring the potential of these methods for quantitative trait improvement in animal breeding.

While our results are promising, further studies are required to fully assess the potential of this method. The primary constraint is the relatively small number of chickens analysed. Thus, further validation is required across different cohort sizes, lines, ages and environments to establish generalizability. In this study, all tibiae were imaged and tested post-mortem because of COVID-19 restrictions, which minimised variation in limb positioning and soft-tissue coverage. Consequently, the image-derived CNN phenotype is likely to have relatively low residual variance compared to an *in vivo* imaging scenario, contributing to the moderate heritability we observed ($h^{2}\approx 0.15-0.16$) and the very high genetic correlation with true breaking strength ($r_{g}\geq 0.90$). In live birds, variation in limb posture, motion and soft tissue composition would be expected to increase environmental noise in the radiographic phenotype, potentially reducing its heritability. However, imaging younger laying hens will increase the expression of genetic differences in skeletal robustness, and longitudinal *in vivo* designs with repeated measures can help to partition permanent environmental and residual sources of variance. Overall, while we anticipate that heritability estimates for image-derived traits may be somewhat lower in live animals than in this controlled postmortem dataset, the underlying genetic covariance with true bone strength should remain high, such that CNN-based scores remain effective selection criteria in practical breeding programmes. We expect data sets to be available in future to test this. However, what can be concluded is that the implementation of deep learning based methods has substantial promise to improve chicken breeding programs for welfare traits and other phenotypes, with such approaches particularly useful in large-scale settings.

As discussed, keel bone integrity is difficult to assess radiographically *in vivo* because of overlying soft tissue and the confounding effects of callus formation on already-damaged keels (Maidin et al., 2025; Riber et al., 2018), whereas tibiotarsal quality has been proposed as a more tractable proxy for overall skeletal robustness (Bishop et al., 2000; Wilson et al., 2023). Recent work has shown that hens with severe keel bone damage have lower tibia breaking strength and density than birds with minimal keel damage (Maidin et al., 2025), supporting a biological link between long-bone quality and keel outcomes. By demonstrating that a CNN applied to tibia radiographs can generate a heritable phenotype that is highly genetically correlated with true tibia breaking strength, our study suggests that image-derived tibial traits could be used in selection programmes to improve general skeletal strength and thereby reduce the incidence and severity of keel bone damage, even if the keel itself cannot be reliably phenotyped at scale.

In summary, in this study, a deep-learning model has been developed that can predict the tibia breaking strength of chickens from their corresponding X-ray images. The model’s predictions show a high and positive genetic correlation with the tibia-breaking strength, thus suggesting that our models could be used as a selection method for tibia-breaking strength during selective breeding. Overall, the non-invasive method of imaging tibia bones that has been demonstrated and the automation of extracting quantifiable estimates of bone quality show considerable promise for improving animal welfare in large-scale breeding programs.

## CRediT authorship contribution statement

**Tanmay Debnath:** Writing – original draft, Methodology, Investigation, Formal analysis, Data curation, Conceptualization. **Peter Wilson:** Writing – review & editing, Validation, Resources, Investigation, Data curation. **Ricardo Pong-Wong:** Writing – review & editing, Methodology, Formal analysis. **Lindsey Plenderleith:** Writing – review & editing, Supervision, Methodology, Conceptualization. **Björn Andersson:** Writing – review & editing, Resources, Project administration, Data curation, Conceptualization. **Matthias Schmutz:** Writing – review & editing, Resources, Project administration, Data curation, Conceptualization. **Ian Dunn:** Writing – review & editing, Supervision, Project administration, Methodology, Funding acquisition, Data curation, Conceptualization. **James G.D. Prendergast:** Writing – review & editing, Supervision, Resources, Project administration, Methodology, Funding acquisition, Conceptualization.

## Disclosures

The authors declare that they have no known competing financial interests or personal relationships that could have appeared to influence the work reported in this paper.

## Appendix Supplementary materials

Image, application 1
