# Supplementary material for: Deep learning can automate chicken tibia-breaking strength quantification to improve animal welfare
Source: Poult Sci. 2026 Jan 30;105(4):106549. doi: 10.1016/j.psj.2026.106549 (PMC12892063; doi:10.1016/j.psj.2026.106549)
Supplement: Supplementary file 1 [file mmc1.docx]

# SUPPLEMENTARY GLOSSARY

Terms related to machine learning in this research have been explained here:

| **Terms** | **Explanation** |
| --- | --- |
| Trial | One complete training–validation run of a model with a particular set of hyperparameters suggested by Optuna. Each trial gives one performance result, used to decide whether that hyperparameter set is good |
| Segmentation | A method to divide the data based on features. In our research, this module has been used to separate the individual tibia bones from the rest of the X-ray images. |
| Regression | A machine learning technique to predict continuous numerical values based on features from the dataset. In our case, the ResNet-50 regression model takes images as input and predicts the corresponding tibia breaking strength as the numerical value |
| Convolutional Neural Network (CNN) | A special kind of deep learning method that takes visual data as input (like images) and extracts relevant features required to make a desired prediction. |
| Encoder blocks | These are specialised blocks in a model (in our case, the U-Net segmentation model) that are capable of extracting rich representations of the input data |
| Decoder blocks | These are specialised blocks in a model (in our case, the U-Net segmentation model) that are capable of producing desired results based on the rich representations of the data stored inside the model as model parameters (or weights) |
| ReLU | To add some non-linearity in the model during the model training, some specialised functions like ReLU are added |
| Downsampling | One of the problems in ML models is that they operate in a very high-dimensional space. It might not be effective (in terms of resources) to compute everything at that space. Thus, to reduce the computational load during model training, methods like downsampling are practised during pooling layers that reduce the dimensions of the representations |
| Upsampling | Specifically, in U-Net models, after features were extracted from the low-dimensional space, they need to be up-scaled to the output matching the input scale. For this, we have to upsample the features such that we get an output that has a similar dimension as compared to the input |
| Dice Loss | A mathematical function used to train segmentation models. It depends on how well the model’s predictions match the ground truth; the higher the match, the lower the loss |
| Epochs | Total number of times a model is run to train it |

# SUPPLEMENTARY FIGURES

**
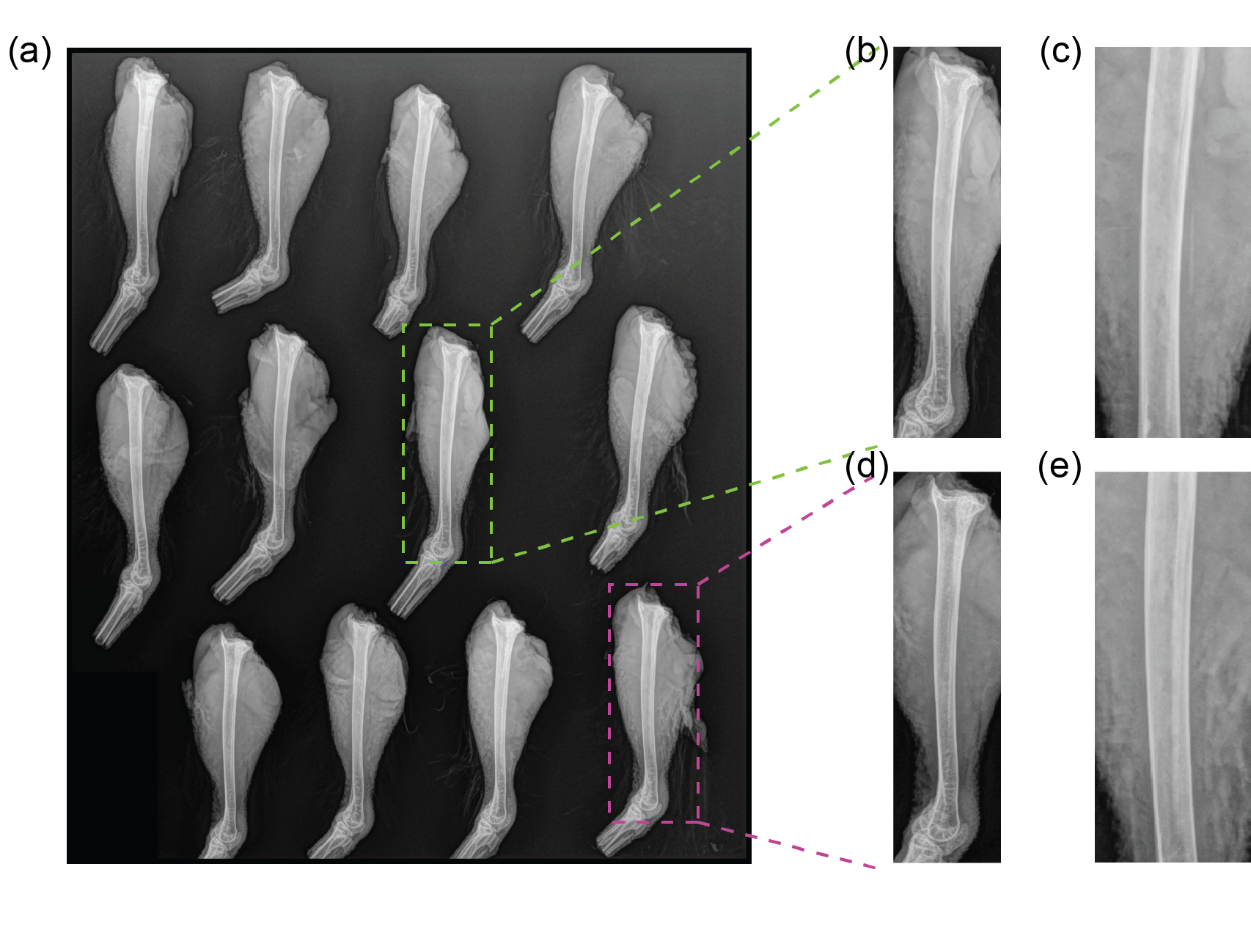
**

**Figure S1. An overview of the data used for model training.** (a) The original dataset contains multiple chicken legs stacked within a single x-ray image. (b) Using the segmentation model we have tagged the individual bone regions [tag ID: 11-6] and extracted a box area enclosing the segmented region. (c) For model training, we have removed the joint areas from the top and bottom regions. (d-e) The same procedure as (b-c) for a different bone region [tag ID: 11-11]


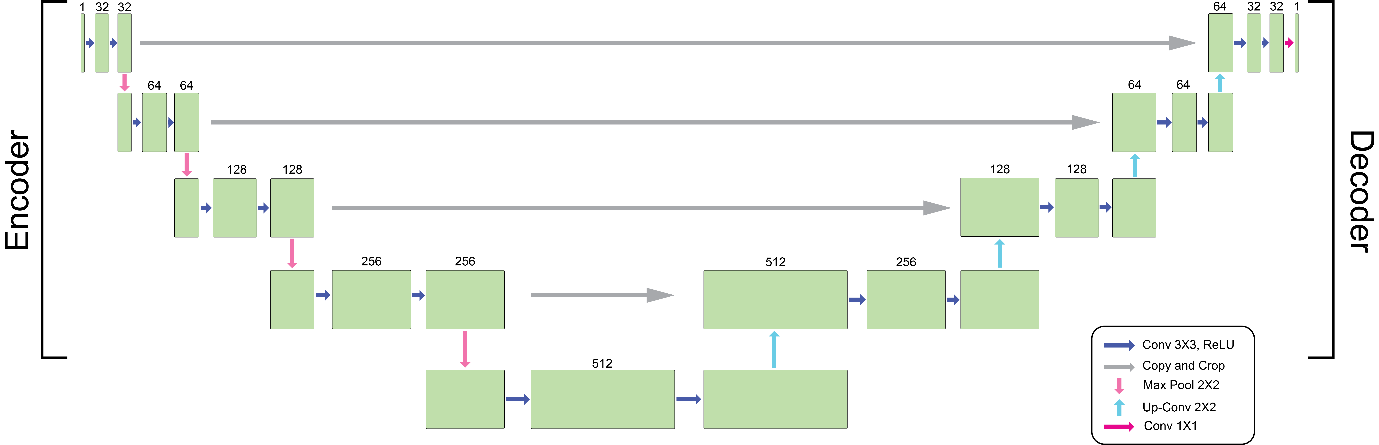


**Figure S2. The U-Net model architecture** (Ronneberger et al., 2015)**.** The encoder-decoder architecture has been used for image-based segmentation tasks.

**
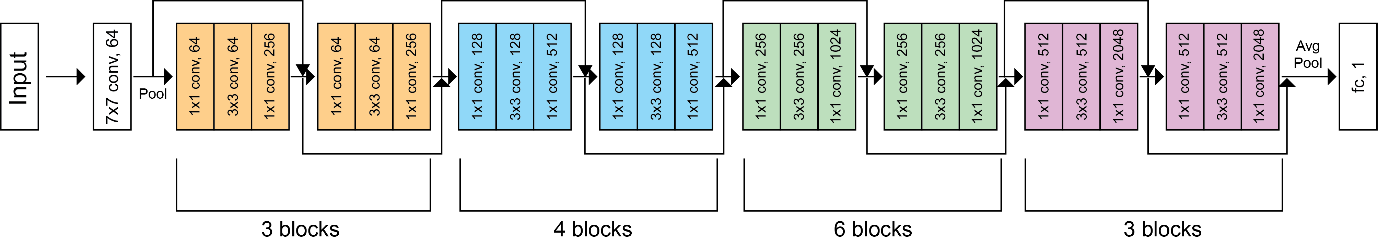
**

**Figure S3. The ResNet-50 model architecture.**


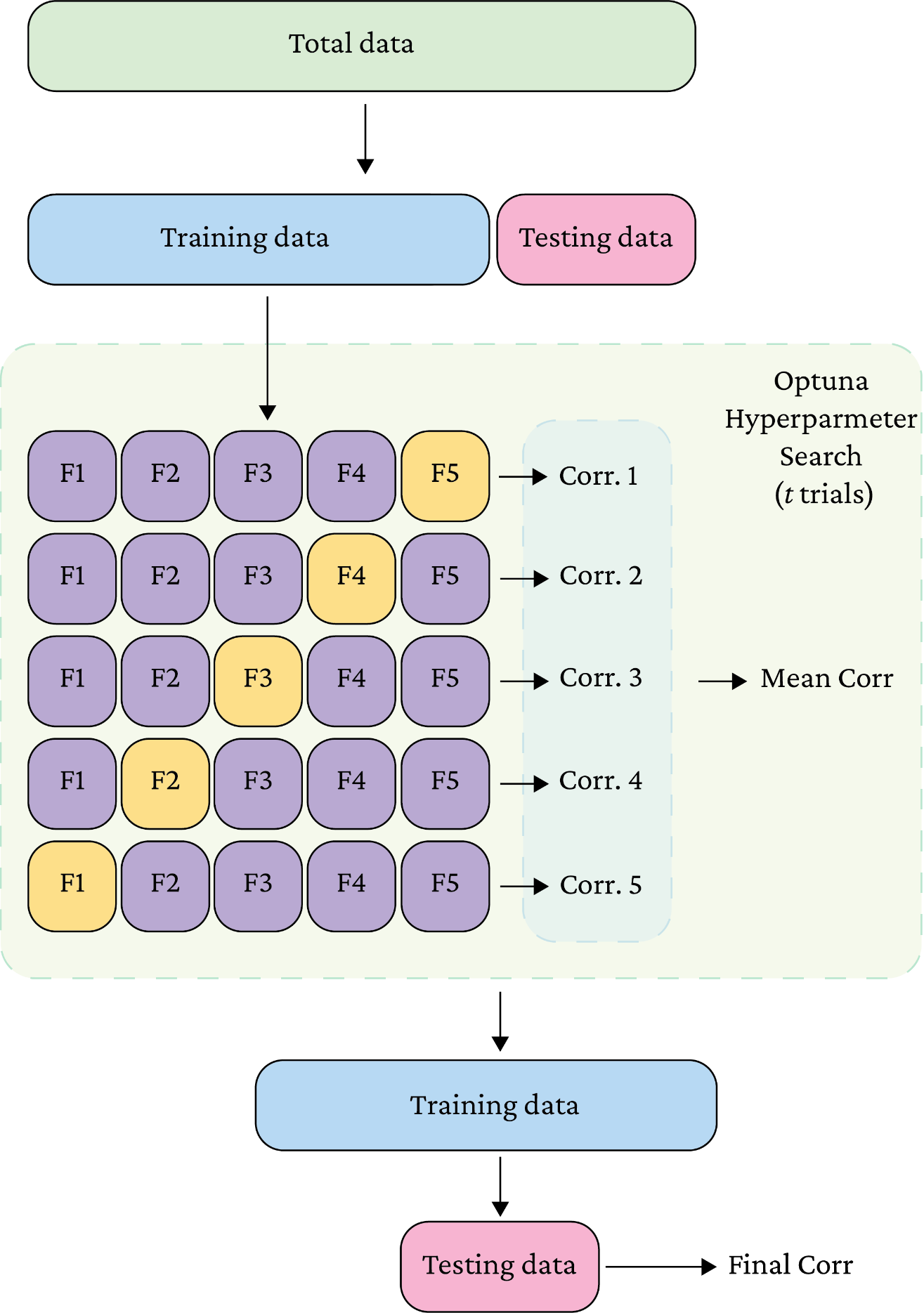


**Figure S4.** **The training scheme implemented for the regression models**. 5-fold cross-validation was employed as an integral part of the model training to ensure that the model is able to generalise its learning irrespective of the different folds of data.


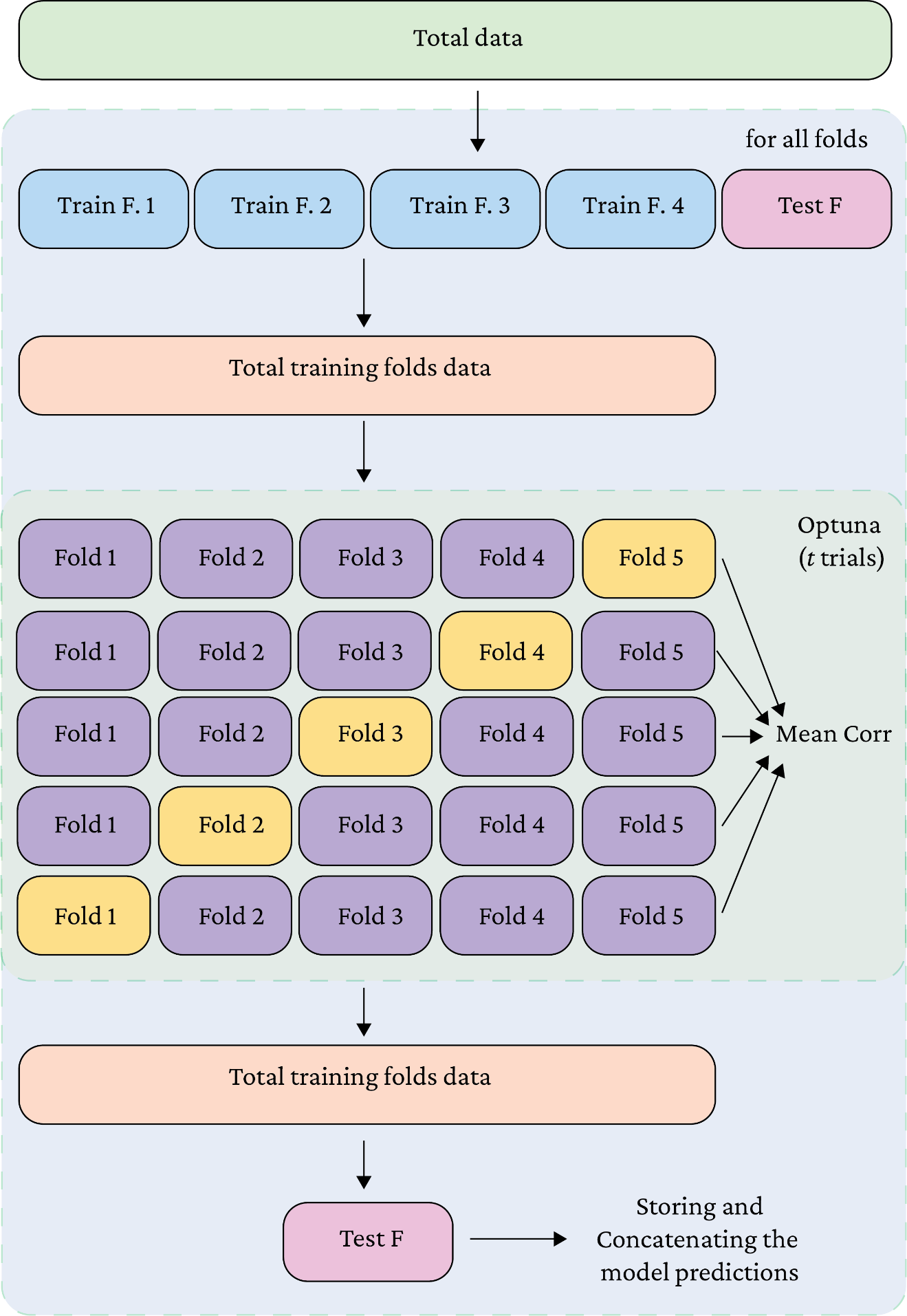


**Figure S5.** **The scheme implemented for obtaining the model predictions for genetic correlation analysis**. 5-fold cross-validation was employed as an integral part of the model training to ensure that the model is able to generalise its learning irrespective of the different folds of data. To obtain the results from the entire daaset, we have split the dataset into multiple folds and kept each fold as a testing dataset at a time such that we can concatenate the prediction one-by-one.

# Supplementary References

Ronneberger, O., Fischer, P., & Brox, T. (2015, 2015). *U-Net: Convolutional Networks for Biomedical Image Segmentation* MICCAI, <https://arxiv.org/abs/1505.04597>
